# A Social Emotional Learning Training Programme in a Poor Rural Primary School in Central China: A Pre-Post Intervention Study

**DOI:** 10.3390/healthcare10112332

**Published:** 2022-11-21

**Authors:** Jiameng Li, Cuiling Ma, Qi Lu, Therese Hesketh

**Affiliations:** 1 Centre for Global Health, Zhejiang University School of Medicine, 866 Yuhangtang Road, Xihu District, Hangzhou 310058, China; 2 The Institute for Global Health, UCL, 30 Guilford Street, London WC1NEH, UK

**Keywords:** social and emotional learning (SEL), pre-post intervention study, school, children, mainland China

## Abstract

Introduction: Many universal school-based social and emotional learning (SEL) programmes in the U.S. and Europe have been found to improve social skills and reduce emotional distress and behaviour problems. The aim of this study is to determine whether an adapted version of the SEL can reduce social, emotional, and behavioural difficulties in children in mainland China, using a pre-post intervention design. Methods: The study was conducted in a primary school in an economically-disadvantaged rural area in Henan province in central China. The intervention consisted of 16 weekly 90-minunte classroom sessions involving all 190 children in the school. Social and emotional problems were assessed pre- and post- intervention using the Chinese version of the Strengths and Difficulties Questionnaire (SDQ). The results suggest that: (1) the programme can reduce children’s peer relationship problems, and that the reduction was sustainable at the two post-intervention assessments; (2) the intervention effects on emotional symptoms or total difficulties in the overall population are very few, but children identified as high risk in the initial assessment benefited from the programme. Conclusions: This is the first published report on the effectiveness of a school-based SEL programme in mainland China. Although the improvement are limited, the programme does benefit some children.

## 1. Introduction

Social-emotional learning (SEL) is the process of developing the self-awareness, self-control, and interpersonal skills that are vital for school, work, and life success. The idea of integrating SEL into the education system across the age range emerged in the early 1990s in the US. SEL was defined as the process of acquiring core abilities to recognise and manage emotions, set and achieve positive goals, feel and show empathy for others, establish and maintain supportive relationships, make responsible decisions, and handle interpersonal situations better [1]. The core goals of SEL programmes are to foster the development of five associated cognitive, affective, and behavioural competencies: self-awareness (e.g., recognising emotions, strengths and limitations), self-management (e.g., regulating emotions and behaviours), social awareness (e.g., taking the perspective of and empathising with others from diverse backgrounds and cultures), relationship skills (e.g., establishing and maintaining healthy relationships), and responsible decision making (e.g., making constructive choices across varied situations) [2]. The United States remains the hub for the development and dissemination of SEL programmes. SEL has been adopted in mainstream education in a number of countries, including Australia, the UK, other European countries, and parts of Asia such as Singapore [3,4].

The results from a meta-analysis of 213 school-based, universal SEL programmes (87% from United States) showed that participants demonstrated improved social skills, fewer behaviour problems, and less emotional distress [5]. A meta-analysis of the effects of universal, school-based social, emotional, and behavioural programmes in 75 studies (80% from North America and 15% from European countries) reported enhancement of social and emotional skills, positive self-image, reduction of antisocial behaviour and mental health problems [6]. A meta-analysis of follow-up effects of school-based, universal SEL interventions involving 97,406 kindergarten to high school students in 82 studies (54% from United States, and others from Australia and European countries) suggested that participants reported improvements in social-emotional skills, attitudes, and well-being regardless of their race or socioeconomic background [7]. This meta-analysis also found no significant difference in the effectiveness between interventions involving predominately low- and working-class students compared with those of middle- and upper-class. A review including 22 studies (mostly from Asian, African, and South American countries) indicated school-based SEL programmes could improve emotional and behavioural wellbeing among children from low- and middle-income backgrounds [8]. Some universal SEL interventions indicated that students from rural low socioeconomic status actually benefit more from the intervention [7]. Stronger intervention effects have been found for students from poor families in improving school achievement and reducing misbehaviour [9].

Very few universal school-based SEL programmes have been conducted in Chinese schools. Published reports on SEL programmes have only been published in Hong Kong. A study among first graders aged 6–7 years in Hong Kong found improvements in emotion regulation and prosocial behaviours after a universal classroom-led SEL programme [4]. A school-based universal SEL programme conducted among seventh-grade Hong Kong children aged 12–13 years reported that the intervention group had less emotional distress, fewer internalizing problems such as depression and anxiety, and attention difficulty [10]. In Chinese culture the pursuit of academic success is widely regarded as a high priority with children and adolescents forced to devote most of their waking hours to studying. Academic success is regarded as essential to higher income and social status [11]. As a result, very little attention is paid to the social and emotional well-being of children. This is despite strong evidence for the importance of confidence, compassion, good communication, good relationships, and emotional stability in order to function well in society and especially in the workplace [12].

In 2011, the Ministry of Education of China and UNICEF initiated a pilot social and emotional learning (SEL) programme in five Chinese provinces (Guizhou, Yunnan, Chongqing, Guangxi, and Xinjiang), based on the British Social and Emotional Aspects of Learning (SEAL) project. Resources were developed based on the pilot experience and were made available after the first COVID-19 lockdown in November 2020, in recognition of the particular need for social emotional training during the COVID pandemic [13]. However, the degree to which the programme was disseminated, as well as the outcomes, are unclear, and there have been no recommendations about incorporating it into the curriculum.

In this study, we piloted an adapted version of the MoE-UNICEF social and emotional learning programme with the aim of testing whether it can be used to reduce social, emotional, and behavioural difficulties in children. We aimed to explore: (1) whether there would be significant improvements in participants’ social emotional wellbeing after the intervention; and (2) whether changes in social, emotional and behaviour problems were related to the initial risk levels of the participants.

## 2. Methods

### 2.1. Participants

The pilot intervention was conducted in a primary school located in an extremely poor village of Henan province in central China, with around 80% of residents with a household income per person of 10,000 yuan or less in 2021, which is much lower than the average level of rural China, i.e., 18,931 yuan [14]. More than 50% of the participants were “left-behind” children, with migrant workers as parents, and usually in the care of grandparents. There is only one class in each grade, a small school by Chinese standards. There are classes only in Chinese, mathematics, and English, with no classes in physical education, music, or painting, which are officially included in the school curriculum. Difficulties in recruiting local teachers to such poor rural schools mean that teachers are brought in on short-term contracts from the nearest city. During exam times, there are no class breaks because the whole day is spent revising for exams. This intensity is driven by competition with other schools for places at better secondary schools.

### 2.2. Procedures

In this pilot trial, a pre-post intervention design was adopted. All school attenders were included in the intervention condition with no control group because of the social emotional needs of the children, logistic challenges, and requirements of the school. All 243 students in the school participated in the intervention, but 43 (18%) first graders were not included in the pre-post analysis because of difficulties understanding the questionnaire; 7 (3%) from higher grades were not included because of missing one of the three assessments, and 3 (1.2%) were excluded because they missed more than two of the intervention sessions. Therefore, 190 (79%) students from 2nd to 6th grade were included in the analysis. Prior to the intervention, emotional and behaviour problems were assessed in January 2021 (Assessment 1). The intervention took place during regular school hours with the title “social skills training” for a whole 4-month semester from September 2021 to January 2022. After the programme was completed, emotional and behaviour difficulties were assessed in all participants in January 2022 (Assessment 2). After 5-month follow-up, the participants were once again assessed with the same questionnaire in June 2022 (Assessment 3).

### 2.3. Adaptation of the SEL Materials

The programme consisted of 16 weekly 90-min class sessions mostly adapted from the MoE-UNICEF social emotional learning resources [13]. The sessions were adapted on the basis of fieldwork and interviews with 30 students, evenly distributed across all grades, and 6 teachers, to fit the developmental, behavioural, and emotional needs of the local students. For example, teachers told us that almost all the children spoke rarely in class to ask or answer questions. To improve this, we used videos of popular cartoons, in which characters demonstrate confidence leading the children to discuss how to be confident. More than half of the children were “left-behind”, by parents who were rural-urban migrant workers, returning typically once or twice per year. These children were mostly looked after by grandparents. At Interviews showed that communication with absent parents was often difficult, so to address this problem, we inserted several stories in the session “express myself” and “empathy training” to help with communicating with parents more comfortably. Several focus groups with children were conducted to identify which type of games from the SEL materials were most suited to the local context, as well as the “warm up” games children preferred in different sessions.

The 16 sessions covered five topics: (1) improvement of self-understanding and help with regulation of emotions; (2) building self-confidence and “feeling good to be me”; (3) help with addressing arguments, communicating effectively, and getting along with others; (4) saying NO to bullying; (5) setting targets and achieving them (Table 1). All sessions involved group discussion, role-play, art activities, storytelling, watching videos, handicrafts, and educational games. A handbook including the objectives and activities of the sessions was written specifically for the programme to guide the volunteers, and a separate booklet including the major content was given to each student.

### 2.4. Selection of the School

We contacted the local education bureau to explain the aims and content of the programme and asked for their support and recommendations for a participating school. After getting approval from the school, a pamphlet was given to the students and their caregivers with detailed information about the programme and a request for their written consent for children’s participation.

### 2.5. Training Volunteers and Implementing the Intervention

The SEL classes were led by a volunteer in each classroom. Six volunteers from the psychology department of a local university were recruited. They were third-year undergraduate students, three of which were majoring in applied psychology and the other three in educational psychology. They did not have experience in leading such intervention groups. They were trained over seven three-hour sessions by the coordinator. The training covered topics such as the general theory and main content of the curriculum, the adaptation of the sessions, and the introduction of the school and students. Volunteers were encouraged to think about daily examples in their experience that were relevant to the teaching of the sessions, and which would facilitate children’s understanding of the topics. They also had the chance to practise and role-playing activities and discussed how to optimise delivery of the sessions in the classrooms.

When the sessions were underway in the classrooms, the coordinator observed and provided support if needed. The coordinator assessed the volunteers’ implementation, including the way they conveyed the core ideas and concepts of the sessions. She gave feedback after each session and helped volunteers make adaptations if problems arose. Since the programme was included in the curriculum, the attendance rate was over 95% at each session.

## 3. Measurement Instruments

Sociodemographic and background information comprised gender, age, grade, number of siblings, the main caregiver, household composition (both parents, one parent, neither parent), family economy status (retrieved from school records and then categorised into three levels-good, fair, poor), and parents’ occupations.

The Chinese version of the Strengths and Difficulties Questionnaire (SDQ) is a screening tool designed for the early detection of social, emotional, and behavioural problems in children and adolescents aged 4 to 16 years [15]. It is very widely used in Chinese research. The SDQ comprises 25 items, divided equally across five subscales: emotional symptoms, conduct problems, hyperactivity, peer relationship problems, and prosocial behaviour. The first four subscales measure potential difficulties and a combined score provide a child’s total difficulties (TD) score with a higher score indicating greater difficulties. The fifth subscale, prosocial behaviour, is measured separately as a ‘strength’, with a higher score indicating better well-being.

### Statistical Analyses

The intervention was carried out in all classes. In order to compare the effects of the intervention on children with different levels of emotional and behaviour difficulties, participants were stratified into low-, moderate- and high-risk groups based on the SDQ total difficulties scores at the first assessment. The upper quartile mean was 7.25 and lower quartile mean was 15, so the SDQ total difficulties score of less than 8 was classified as low risk, 8–14 moderate risk, and 15 and over high risk.

The impact of the programme on emotional and behaviour problems was evaluated by comparing scores on the SDQ subscales in the three risk groups at three time points: before the intervention (January 2021), immediately after completion of the intervention (January 2022), and 5-month follow-up (June 2022).

Data analysis was performed with SPSS 24.0. First, we generated descriptive statistics on the sociodemographic information. Second, we used Pearson’s chi-square tests to examine the association between gender and sociodemographic information. Third, repeated measures analysis of variance (ANOVA) was conducted with three risk groups (low, moderate, high) as between-subjects factor and the SDQ components as repeated measures (pre-intervention, post-intervention, and follow-up assessment). Fourth, one-way repeated measures analysis of variance (ANOVA) was conducted in different risk groups separately with SDQ-difficulties as repeated measures (pre-intervention, post-intervention, and follow-up assessment).

## 4. Results

The sociodemographic characteristics of the participants who completed all three assessments are presented in Table 2. The chi-squared test for categorical variables revealed no significant differences between genders. Around 38% of the participants were mainly looked after by grandparents and around half did not live with both parents.

### 4.1. Intervention Effects on SDQ Components

There was an effect of the time point on peer problem (η^2^ = 0.15), which means there were significant differences among the peer problem scores in the three assessments (Table 3 and Table 4). Peer problem scores lowered at Assessment 2 (*p* < 0.001) and Assessment 3 (*p* < 0.001) compared to Assessment 1, while there were no differences between Assessment 2 and Assessment 3. This suggested that peer problems decreased from pre- to post-assessments, and that the reduction was sustainable at the two post-intervention assessments. There was an effect of the time point on hyperactivity (η^2^ = 0.06), which means there were significant differences in the scores of hyperactivity in the three assessments (Table 3 and Table 4). There was no effect of the time point on SDQ total difficulties, conduct problems, emotional symptoms, or prosocial behaviours. Meanwhile, there was a time * risk group interaction effect of the SDQ-total difficulties (η^2^ = 0.18), conduct problems (η^2^ = 0.1), emotional symptoms (η^2^ = 0.11) and hyperactivities (η^2^ = 0.11). This suggested that the effect of the time point was different in the three risk groups of total difficulties, conduct problems, emotional symptoms and hyperactivities.

### 4.2. Intervention Effects on Participants of Different Risk Groups

The intervention effects of the SDQ-total difficulties, conduct problems, emotional symptoms, and hyperactivities in three risk groups are presented in Table 5.

In the high-risk group, there was an effect of the time point on SDQ-total difficulties (η^2^ = 0.25) which lowered at Assessment 2 (*p* < 0.001) and Assessment 3 (*p* < 0.001) compared to Assessment 1, with no difference between Assessments 2 and 3. This suggested that in the high risk group, the total difficulties decreased from pre- to post-assessments, and that the reduction was sustainable at the two post-intervention assessments. In the moderate risk group, there was no effect of the time point on SDQ-total difficulties (η^2^ = 0.027). In the low-risk group, there was an effect of the time point on SDQ-total difficulties (η^2^ = 0.37), which increased at Assessment 2 (*p* < 0.001) and Assessment 3 (*p* < 0.001) compared to Assessment 1, with no difference between Assessments 2 and 3. This suggested that in the low risk group, total difficulties increased from pre- to post-assessments, and that the increase was sustainable at the two post-intervention assessments.

In the high-risk group, there was an effect of the time point on conduct problems (η^2^ = 0.12), which lowered at Assessment 2 (*p* = 0.016) compared to Assessment 1, with no difference between Assessments 3 and 1 (*p* = 0.07). This suggested that in the high-risk group, conduct problems decreased from baseline to post-intervention, but increased at the 5-month follow-up. In the moderate risk group, there was no effect of the time point on conduct problems (η^2^ = 0.026). In the low-risk group, there was an effect of the time point on conduct problems (η^2^ = 0.26), which increased at Assessment 2 (*p* = 0.002) and Assessment 3 (*p* < 0.001) compared to Assessment 1, with no difference between Assessments 2 and 3. This suggested that in the low risk group conduct problems increased from pre- to post-assessments, and that the increase was sustainable at the two post-intervention assessments.

In the high-risk group, there was an effect of the time point on emotional symptoms (η^2^ = 0.16), which lowered at Assessment 2 (*p* = 0.03) and Assessment 3 (*p* < 0.001) compared to Assessment 1, with no difference between Assessments 2 and 3. This suggested that in the high risk group, emotional symptoms decreased from pre- to post-assessments, and the reduction was sustainable at the two post-intervention assessments. In the moderate risk group, there was no effect of the time point on emotional symptoms (η^2^ = 0.016). In the low-risk group, there was an effect of the time point on emotional symptoms (η^2^ = 0.25), which increased at Assessment 2 (*p* = 0.008) and Assessment 3 (*p* < 0.001) compared to Assessment 1, with no difference between Assessments 2 and 3. This suggested that in the low risk group, emotional symptoms increased from pre- to post-assessments, and that the increase was sustainable at the two post-intervention assessments.

There was no effect of the time point on hyperactivities in the high-risk group (η^2^ = 0.036) and moderate risk group (η^2^ = 0.037). In the low risk group, there was an effect of the time point on hyperactivities (η^2^ = 0.42), which increased at Assessment 2 (*p* < 0.001) and Assessment 3 (*p* < 0.001) compared to Assessment 1, with no difference between Assessment 2 and 3. This suggested that in the low risk group hyperactivities increased from pre- to post-assessments, and that the increase was sustainable at the two post-intervention assessments.

## 5. Discussion

To our knowledge, this is the first study to test the effectiveness of a universal school-based SEL programme for primary school children in mainland China. The key findings are: (1) the programme can reduce children’s peer relationship problems and the reduction was sustainable at the two post-intervention assessments; (2) no intervention effect was found on emotional symptoms, conduct problems or hyperactivity in the whole participants, but the high-risk children benefited from the programme.

The peer problems reduced after the intervention and the reduction was sustainable at the two post-intervention assessments. This may have been because of the focus of our programme, which was on positive communication, collaboration, and problem-solving skills within the classroom context with the aim of enhancing children’s ability to understand their differences, be more empathic, and resolve conflicts effectively. Children’s peer communication skills can be improved with an increase in mutual understanding and acceptance [16]. The role plays are reported to be effective in fostering perspective taking and empathy [17]. In our study, the pre-prepared role plays scenarios relevant to the school setting are widely used through all sessions, for example, role plays dealing with how to communicate with students who have been making jokes on you that makes you embarrassed.

The intervention did not improve emotional symptoms, conduct problems, or hyperactivity in the whole participants. This may have been due to the relative brevity of the intervention, which spanned just 4 months. It is hard to acquire the skills that can have a consequential influence on emotional well-being or hyperactivity in such a short time [18]. There is also a problem that the programme put too much responsibility on the children to integrate the newly learned skills and strategies into their behaviours; for example, a common strategy in the programme involves teaching children how to modify unhelpful thoughts into helpful thoughts, which activate positive behaviours. An alternative approach would be to train schoolteachers to better recognise and reward helpful thoughts expressed by the children. Consequently, an approach where teachers are substantially trained in such core practice elements instead of only receiving a short introduction of the general idea of the programme may enhance the preventive effect [19]. Another possible explanation for this lack of significance is the delayed effect, which means the effectiveness cannot be found after a short period of the intervention, but can be seen after extended intervals for participants to practise, apply and consolidate skills [20]. So further assessments with longer follow-ups are required.

Since this is a universal study, the lack of effectiveness could also be attributed to the selection of the sample. In the general population of children, there will always be a number of subjects who do not present any risk of social emotional problems [21]. In our study, the emotional symptoms, conduct problems, and hyperactivity levels were quite low for the low-risk children at the pre-intervention assessment, and these difficulties even increased at post-intervention assessments but were still within the normal range. Room for improvement is quite small for such low-risk children [22]. However, the effectiveness of the reduction of total difficulties and emotional symptoms at post intervention and 5-month follow-up could be observed in high-risk children in our study. Similar results had been found from other studies, that is, the children most responsive to the effects of the social emotional training programmes were the ones with higher levels of emotional and behavioural difficulties at pre-intervention assessment [23,24].

There were a number of limitations in this study. A primary one was the lack of a control group. It is possible that the improvements in children’s social and emotional outcomes were due to other factors and not the programme, for example, “natural” developmental progression in the 4-month period in which the intervention was implemented. Therefore, longer term follow-up is needed. Second, the results were based on children’s self-reports and, ideally, future research should include data from other informants, such as parents, teachers, and caregivers. Third, given that the programme was conducted in just one school in a poor rural area of central China, the findings cannot be generalised.

## 6. Conclusions

This intervention was welcomed by the school, incorporated into the curriculum, and was cheap to implement, making it potentially sustainable. The programme is easy to implement with the guidance of the handbook. It can be delivered to school children in regular school hours and could be integrated into the classroom curriculum, ensuring a high attendance rate and retention.

To our knowledge, this is the first published report of the effectiveness of a universal school-based SEL programme in mainland China. Although the findings are preliminary, they support the evidence for the effectiveness of the programme to improve peer relationships among primary school children. With the support of the local Education Bureau, schools could incorporate the SEL programme into their formal curriculum, as has been the case in a number of countries [3,25]. Schools in Hong Kong have been urged by the governmental Education Bureau to regard the all-round development and social emotional wellbeing of their students as major educational aims, with the incorporation of SEL in the curricula [4]. Our study shows that the SEL programme is cheap to implement and potentially sustainable in poor rural areas in China. Local people who are interested in and capable of delivering the intervention sessions should be trained and employed to deliver the programme. Implementation should be the responsibility of the local government education department.

Our findings show that future studies should: (1) train schoolteachers and other school staff in SEL skills so that children’s positive behaviours can be strengthened outside of SEL classes. SEL skills are also needed in playgrounds and in lunchrooms, and thus, school staff, who have interaction with children should be trained in such skills; (2) shorten the duration of each session to 40–50 min. Although there is a 10-min class break in the middle of the 90-min session, most children could not focus well in the second half, which might compromise the effectiveness of intervention sessions. (3) A more intense programme could be provided for high-risk children.

## Figures and Tables

**Table 1:** Content of the intervention Sessions.

Topic	Session Title
1. Learn about emotions	(1) Different and complex emotions
	(2) How emotions influence behaviours
	(3) Emotion management
2. Good to be me	(1) Be confident and aware of my strengths
	(2) Express myself bravely and stay true to myself
	(3) Relax and calm down
3. Get along with others	(1) Show kindness and care for others
	(2) Empathy training and put yourself in other’s place to understand the differences
	(3) Learn to resolve conflicts
	(4) Take responsibility in collaboration
4. Say NO to bullying	(1) Bullying and being bullied
	(2) Empathise with bullying victims
	(3) What can we do if involved in bullying
	(4) How to stop bullying
5. Move toward your goals	(1) Make a goal and a plan
	(2) Strategies of overcoming difficulties such as boredom, tiredness, and procrastination

**Table 2:** Sociodemographic and background information of participants at Assessment 1.

	Total (n = 190)	Male (n = 81)	Female (n = 109)	Chi-Square	*p*
Age (mean = 9.21, SD = 1.1)				0.124	0.725
7–9	120 (63.2)	50 (61.7)	70 (64.2)		
10–12	70 (36.8)	31 (38.3)	39 (35.8)		
Number of siblings (mean = 1.91, SD = 1.038)				3.25	0.517
0	11 (5.8)	5 (6.3)	6 (5.6)		
1	76 (40)	36 (45)	40 (37)		
2	72 (37.9)	25 (31)	47 (43.5)		
3	22 (11.6)	10 (12.5)	12 (11)		
4	7 (3.7)	4 (5)	3 (2.8)		
The main carer				0.83	0.66
Grandparents	72 (37.9)	33 (41.3)	39 (36.1)		
Father	8 (4.2)	4 (5)	4 (3.7)		
mother	108 (56.8)	43 (53.8)	65 (60.2)		
Household composition				0.905	0.636
Both parents	93 (48.9)	37 (46.8)	56 (51.9)		
One parent	55 (28.9)	23 (29.1)	32 (29.6)		
Neither parent	39 (20.5)	19 (24.1)	20 (18.5)		
Family economic status				2.542	0.281
Above average	60 (31.6)	26 (40.6)	34 (35.8)		
Average	89 (46.8)	32 (50)	57 (60)		
Below average	10 (5.3)	6 (9.4)	4 (4.2)		

**Table 3:** Descriptive statistics for SDQ components, mean (SD).

	Total	Low Risk (n = 43)	Moderate Risk (n = 83)	High Risk (n = 46)
SDQ-total difficulties				
Assessment 1	11.9 (5.6)	5.1 (1.8)	11.5 (1.9)	19.0 (3.8)
Assessment 2	11.2 (6.0)	8.7 (5.2)	10.6 (5.6)	14.7 (6.0)
Assessment 3	12.1 (5.5)	10.0 (4.4)	11.7 (5.5)	14.8 (5.4)
SDQ-peer problem				
Assessment 1	3.7 (1.7)	2.2 (1.2)	3.9 (1.4)	4.9 (1.5)
Assessment 2	2.5 (2.0)	1.9 (1.8)	2.5 (1.7)	3.1 (2.4)
Assessment 3	2.6 (1.8)	1.8 (1.2)	2.7 (1.7)	3.2 (2.1)
SDQ-conduct problem				
Assessment 1	1.6 (1.5)	0.5 (0.7)	1.4 (1.0)	3.1 (1.7)
Assessment 2	1.6 (1.6)	1.2 (1.3)	1.5 (1.5)	2.2 (1.7)
Assessment 3	1.9 (1.4)	1.5 (1.2)	1.7 (1.4)	2.4 (1.6)
SDQ-emotional symptoms				
Assessment 1	3.6 (2.4)	1.4 (1.3)	3.4 (1.5)	6.1 (2.1)
Assessment 2	3.7 (2.5)	2.5 (2.1)	3.6 (2.5)	5.0 (2.4)
Assessment 3	3.9 (2.3)	3.2 (2.2)	3.8 (2.4)	4.7 (2.1)
SDQ-hyperactivity				
Assessment 1	3.0 (2.0)	1.1 (1.1)	2.9 (1.5)	4.9 (1.5)
Assessment 2	3.4 (2.3)	3.1 (2.3)	3.9 (2.2)	4.3 (2.2)
Assessment 3	3.8 (2.1)	3.4 (1.9)	3.6 (2.1)	4.6 (2.0)
SDQ-strength (prosocial behaviour)				
Assessment 1	7.9 (1.8)	8.7 (1.6)	7.8 (1.7)	7.3 (1.7)
Assessment 2	7.8 (2.0)	8.0 (1.9)	7.7 (2.1)	7.9 (1.9)
Assessment 3	7.5 (2.0)	7.9 (2.0)	7.6 (1.8)	7.1 (2.2)

**Table 4:** Two-way repeated measures ANOVA for assessing the differences of SDQ scores in three time points by risk groups.

Effect	MS	df	F	*p*	η^2^
SDQ-total difficulties					
Time	34.4	1.8	2.14	0.124	0.01
Risk group	2335.8	2.0	65.11	<0.001	0.44
Time * risk group	296.7	3.7	18.48	<0.001	0.18
SDQ-peer problem					
Time	72.8	1.8	29.0	<0.001	0.15
Risk group	107.4	2.0	26.63	<0.001	0.24
Time * risk group	9.6	3.7	3.83	0.006	0.04
SDQ-conduct problem					
Time	3.1	1.9	2.31	0.10	0.01
Risk group	85.1	2.0	26.7	<0.001	0.24
Time * risk group	11.9	3.8	8.87	<0.001	0.1
SDQ-emotional symptoms					
Time	3.6	2.0	1.26	0.29	0.007
Risk group	283.3	2.0	36.15	<0.001	0.3
Time * risk group	30.6	4.0	10.66	<0.001	0.11
SDQ-hyperactivity					
Time	31.7	1.9	11.15	<0.001	0.06
Risk group	154.3	2.0	26.99	<0.001	0.24
Time * risk group	28.1	3.8	9.89	<0.001	0.11
SDQ-strength (prosocial behaviour)					
Time	7.6	2.0	3.15	0.04	0.02
Risk group	19.9	2.0	3.34	0.04	0.04
Time * risk group	4.4	4.0	1.83	0.12	0.02

Note. “*” refers to the interaction of time and risk group.

**Table 5:** One-way repeated measures ANOVA of SDQ components in three risk groups.

Effect	MS	df	F	*p*	η^2^
High risk group					
SDQ-total difficulties					
Time	318.4	1.8	14.76	<0.001	0.25
SDQ-conduct problem					
Time	12.8	1.6	5.92	0.007	0.12
SDQ-emotional symptoms					
Time	24.1	2.0	8.76	<0.001	0.16
SDQ-hyperactivity					
Time	4.26	2.0	1.66	0.196	0.04
Moderate risk group					
SDQ-total difficulties					
Time	36.55	1.8	2.28	0.112	0.03
SDQ-conduct problem					
Time	2.6	2.0	2.13	0.122	0.03
SDQ-emotional symptoms					
Time	4.1	2.0	1.36	0.261	0.02
SDQ-hyperactivity					
Time	10.5	1.8	3.1	0.053	0.04
Low risk group					
SDQ-total difficulties					
Time	272.2	2.0	24.68	<0.001	0.37
SDQ-conduct problem					
Time	12.7	2.0	15.1	<0.001	0.26
SDQ-emotional symptoms					
Time	37.0	2.0	14.0	<0.001	0.25
SDQ-hyperactivity					
Time	66.8	2.0	30.8	<0.001	0.42
